# The effect of feedstock concentration on the crystal phase, morphology, and optical properties of WO_3_ nanostructures†

**DOI:** 10.1039/d4ra07112c

**Published:** 2025-01-22

**Authors:** Mohsen Zafari, Fatemeh Shariatmadar Tehrani, Seyed Hossein Hosseini Shokouh, Alexey Popov, Krisztian Kordas

**Affiliations:** a Faculty of Physics, Semnan University P.O. Box 35195-363 Semnan Iran f_tehrani@semnan.ac.ir; b Microelectronics Research Unit, Faculty of Information Technology and Electrical Engineering, University of Oulu P.O. Box 4500 FIN-90014 Oulu Finland; c Department of Electronics and Nanoengineering, Aalto University Tietotie 3 FI-02150 Finland seyed.hosseinishokouh@aalto.fi; d VTT Technical Research Centre of Finland Kaitoväylä 1 FI-90590 Oulu Finland

## Abstract

In this study, the effect of feedstock concentration on the synthesis of WO_3_ nanostructures in a one-step hydrothermal process was investigated. According to our experiments, when titrating aqueous Na_2_WO_4_·2H_2_O with HCl solutions of different concentrations to a constant pH of 1.5, after identical hydrothermal treatments at 180 °C, the morphology, crystal size and phase composition as well as the optical properties of the products could be tuned. Furthermore, by applying Na_2_SO_4_ in the precursor solution, we get a further degree of freedom to alter the structural and optical properties of the products. The precursor concentration dependent optical band gap energy values (that vary from ∼2.5 eV to ∼2.9 eV) are in good agreement with the associated hexagonal (from 32.7% to 65.5%) and orthorhombic (from 67.3% to 34.5%) phases of nanostructured tungsten oxide products. The calculated Urbach energies can be well explained by the oxygen deficient crystals. The latter is also coherent with measured photoluminescence (multiple peaks between 400 and 580 nm) that indicate abundant defect states in the tungsten oxide products.

## Abstract 2

Tuning acid molarity at constant pH decreases the feedstock concentration and alters the morphology and crystal phases of WO_3_.

## Graphical abstract

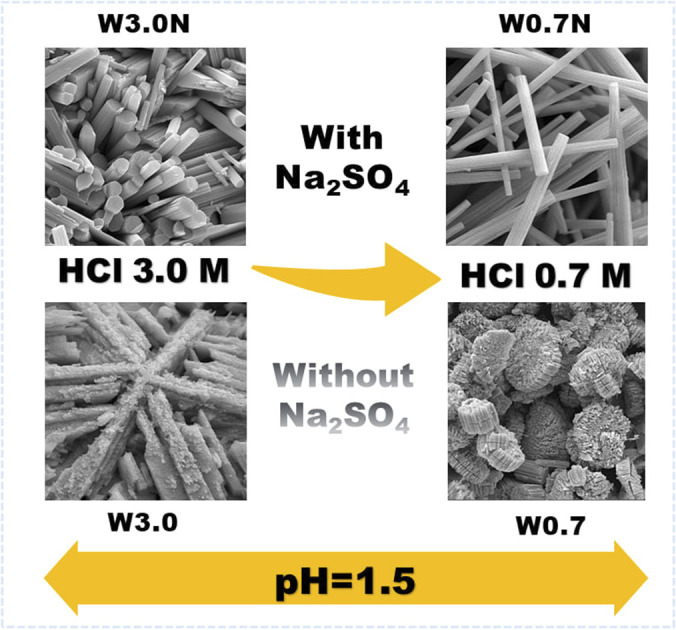

## Introduction

In recent years, tungsten oxides have been widely investigated owing to their tuneable bandgap along with the large variety of possible oxidation states, stoichiometries, crystal phases, and microstructures, and consequently, distinct optical and electrical properties, that have been exploited in applications including transistors,^1,2^ solar cells and optoelectronic devices,^3–7^ photocatalyst^8,9^ and gas sensors.^10–14^

A large variety of morphologies and phases of WO_3_ nanostructures have been synthesized by methods including thermal oxidation of tungsten,^15,16^ physical^17,18^ and chemical vapor deposition^19,20^ sol–gel^21,22^ as well as by hydrothermal reactions.^23–34^ Of these, the latter is especially facile and has gained significant popularity as it is a low-cost single-step process with potentially high-volume production and yield. Furthermore, a number of factors in hydro/solvothermal synthesis affect the morphology and phase of WO_3_ nanostructures, such as the feedstock concentration^29^ and solvent mixture composition,^31^ use of directing agents (*e.g.* ammonium acetate, oxalic acid, surfactants),^25,26^ pH of the precursor,^27–29^ and temperature,^30^ providing a large degree of freedom to engineer the properties of WO_3_ nanostructures. For example, Nagy *et al.* found that an increase in pH causes changes in morphology from nanoplates to nanowires, along with changing the crystal phase from monoclinic to hexagonal.^28^ Shirke and Mukherjee have shown that it is not only the pH but also addition of Na_2_SO_4_ and K_2_SO_4_ that can lead led to different crystalline phases (orthorhombic, hexagonal and monoclinic) and morphologies (nanorods, cocoons, fishbones, among others; more details in Table S1†).^34^ Ou *et al.* synthesized five types of WO_3_·*n*H_2_O crystals orthorhombic nanoplates, rectangular monoclinic nanosheets, orthorhombic microspheres, hexagonal nanorods, and bundle-like hierarchical structures by adjusting the amount of H_2_SO_4_ and the reaction temperature.^30^

Although Zheng *et al.*^29^ have studied the effects of growth conditions including the precursor solutions concentration (H^+^ and Na_2_WO_4_), the additive NaCl, reaction time and growth temperature on the micro and crystal structure of WO_3_, the effect of feedstock concentration while keeping the precursors pH constant and the additives on optical properties has not been widely explored yet. Therefore, in our study, we synthesize tungsten oxide products from solution, in which the pH is kept at a constant value (pH ∼ 1.5), while using different concentrations of HCl solutions for the titration (0.3 M, 0.7 M and 3.0 M), hence altering the concentration of the tungstate precursor.

## Experimental

### Materials and synthetic procedures

WO_3_ nanostructures were synthesized by modifying the hydrothermal route reported earlier by Szabó and co-workers.^23^ 2.5 g of sodium tungstate dihydrate Na_2_WO_4_·2H_2_O (99.9%) was dissolved in 80 mL of distilled water and then titrated with HCl (aq. 3.0 M and 0.7 M) until pH ∼ 1.5 (samples labeled as W3.0 and W0.7). Likewise, 2.5 g of Na_2_WO_4_·2H_2_O and 3.0 g of sodium sulfate (Na_2_SO_4_, 99.2%) were dissolved in 80 mL of distilled water, and then titrated to pH ∼ 1.5 with 3.0 M and 0.7 M HCl (labeled as W3.0N and W0.7N). Each precursor was then placed in an autoclave of 200 mL volume and kept at 180 °C for 48 hours (in a glass vessel of a Teflon-lined steel autoclave, without stirring). Following this, the products were collected by centrifugation and washed with distilled water, and ethanol and dried at 60 °C in air, and then finally were subjected to analyses including BET, FESEM, FT-IR, Raman and PL spectroscopy (see details in Characterization methods).

A further batch of samples were synthesized using similar procedures as before but, in a Teflon-lined steel vessel of ∼800 mL (without stirring). Another difference compared to the previous batch was the precursors were made using de-ionized water, and a fifth sample was also prepared by titrating the tungstate solution with 0.3 M HCl. The products were collected, vacuum-filtered and washed until pH > 6.1 and then dried at 80 °C before XRD and DRS analyses (see details in Characterization methods).

### Characterization methods

The microstructure of the products was studied by field-emission scanning electron microscopy (FESEM, Tescan MIRA3). Textural properties of the products were analyzed by N_2_ adsorption–desorption (Belsorp-mini II) measurements. Brunauer–Emmett–Teller (BET) model was applied to evaluate the specific surface area (*S*_BET_) of the samples. The nature of chemical bonds in the products was assessed by Fourier transform infrared spectroscopy (FTIR, Shimadzu, model 8400S, from 400–4000 cm^−1^) and Raman microscopy (WITec alpha 300 RA^+^ system equipped with a Newton Andor EMCCD and a 100× CF Plan Nikon objective, NA = 0.95, and 532 nm CW laser source). Photoluminescence (PL) spectra were recorded with an Avaspec 2048 TEC instrument, at 370 nm excitation. The band gap and Urbach energy of the samples were determined from UV-Vis diffuse reflectance spectroscopy (DRS) measurements (Agilent Cary 5000, 175–3300 nm). Crystal phase composition of the products was assessed using X-ray diffraction (Rigaku, Miniflex 600 with a Co anode at 40 kV and 15 mA, 2*θ* step size of 0.02 degree).

## Results and discussion

### Microstructure

**Fig. 1:**
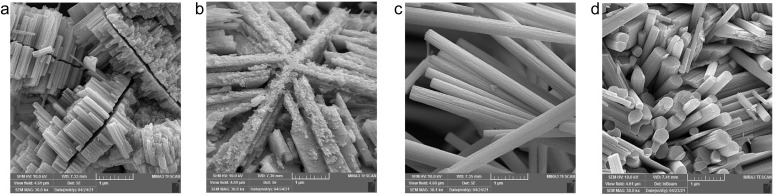
FESEM images of WO_3_ nanostructures prepared using HCl solutions with different acid molarity (a) W0.7 (0.7 M HCl, sunflower islands), (b) W3.0 (3 M HCl, frost-like), (c) W0.7N (0.7 M HCl with Na_2_SO_4_, nanorod bundles), and (d) W3.0N (3 M HCl with Na_2_SO_4_, cohesive nanorod bundle).

Fig. 1 shows FESEM images of the morphology of the synthesized samples. While the W3.0 product (Fig. 1b) has a frost-like morphology, consisting of intercrossed nano-rectangular features, adding Na_2_SO_4_ leads to the formation of cohesive nanorod bundles in W3.0N (Fig. 1d). By lowering the feedstock concentration (W0.7), the nanorods grow anisotropically and symmetrically on both sides of a gap to form sunflower island-like structures (Fig. 1a, and S1†). In 2021, Yin and co-workers reported the formation of a morphology similar to our W0.7, which has significant photocatalytic activity and stability due to its unique transverse longitudinal structure.^32^ In addition, the presence of Na_2_SO_4_ in the precursor (W0.7N and W3.0N) resulted in the formation of highly crystalline nanorod bundles (Fig. 3c and d). The diameter/length of the bundles for the samples W0.7N and W3.0N are in the range of 100–570 nm/1–7 μm and 130–780 nm/0.35–1.5 μm, respectively. In their study,^29^ Zheng *et al.* showed that the density and length of hydrothermally grown WO_3_ nanograss/wire films grown on indium-doped tin oxide surface correlated strongly with the concentration of the sodium tungstate precursor and sodium chloride additive in the solution. Even a very small amount NaCl (2.5 mM) could significantly enhance both the length and diameter of their elongated nanoproducts. Although we use significantly higher concentrations of the Na_2_SO_4_ (0.25 M in W3.0N and 0.20 M in W0.7) in our experiments, the difference between the products with or without the additive was not so striking other than the shape of the crystals (sunflower and frost-like structures without the additive and nanorods with additive). The promoted formation of rod-shaped hexagonal phase products in the presence of high concentration of Na_2_SO_4_ was also reported by Huang *et al.*^33^

The BET specific surface area of nanostructures determined from N_2_ adsorption–desorption isotherms (Fig. S2†) is small (∼20 m^2^ g^−1^ or lower), which is reasonable considering the large crystals observed by FESEM. Furthermore, EDX analysis of prepared samples (Fig. S3†) indicates the structures are oxygen deficient.

### FTIR and Raman spectroscopy

**Fig. 2:**
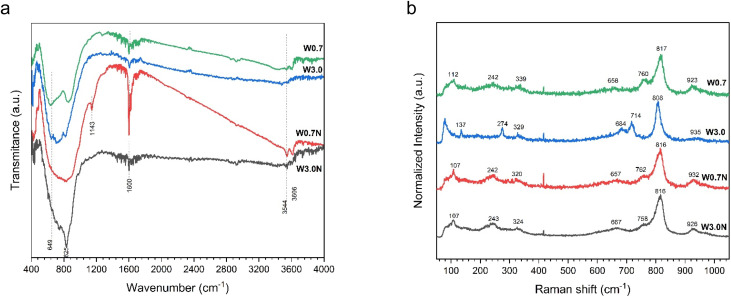
(a) FTIR spectra and (b) Raman spectra of W0.7, W3.0, W0.7N, and W3.0N powder at room temperature.

The FTIR spectra of the prepared products can be seen in Fig. 2a. The stretching and bending vibrations of the water molecule can be easily identified at about 3606, 3544, and 1600 cm^−1^, respectively.^35–37^ The observed shoulders at 649 and 825 cm^−1^ can be assigned to *ν*(O–W–O) and *ν*(W–O) stretching modes, respectively.^36,38^ The absorption peak at 1143 cm^−1^ can be attributed to *δ*(OH) stretching vibration in W–OH, which is unique to W0.7N.^39^ Additionally, the spectra of W0.7, W3.0, and W3.0N samples show the contribution of some sharp peaks in the absorption band from 500 to 900 cm^−1^ (Table S2†). However, in the case of W0.7N, they form only a broad band in this region, which has a higher width compared to the other samples, and the presence of different vibration modes could not be distinguished (see Table S2† for more details).

In contrast to FTIR spectra, the Raman spectra displayed in Fig. 2b indicate some differences, probably stemming from simultaneous presence of different crystal phases, particle size and defects.^40^ For instance, the primary vibration modes of WO_3_ nanopowders are located in the wavenumber range of 200–400 cm^−1^, which is associated with the stretching of *δ*(O–W–O).^41^ The range of 650–820 cm^−1^ is ascribed to the *ν*(O–W–O) with the peak at 714 cm^−1^ assigned to *ν*(W–O) and 760 cm^−1^ attributed to the antisymmetric stretch of transition metal oxide bond.^40–42^ Additionally, the range of 900–950 cm^−1^ refers to the stretching mode of W=O.^40–42^ Although, the peak at 808 cm^−1^ can be ascribed to the monoclinic structure of WO_3_, it has also been assigned to orthorhombic or hexagonal phases.^43^ The detailed peak assignment is provided in Table S3.†

### Photoluminescence spectroscopy

**Fig. 3:**
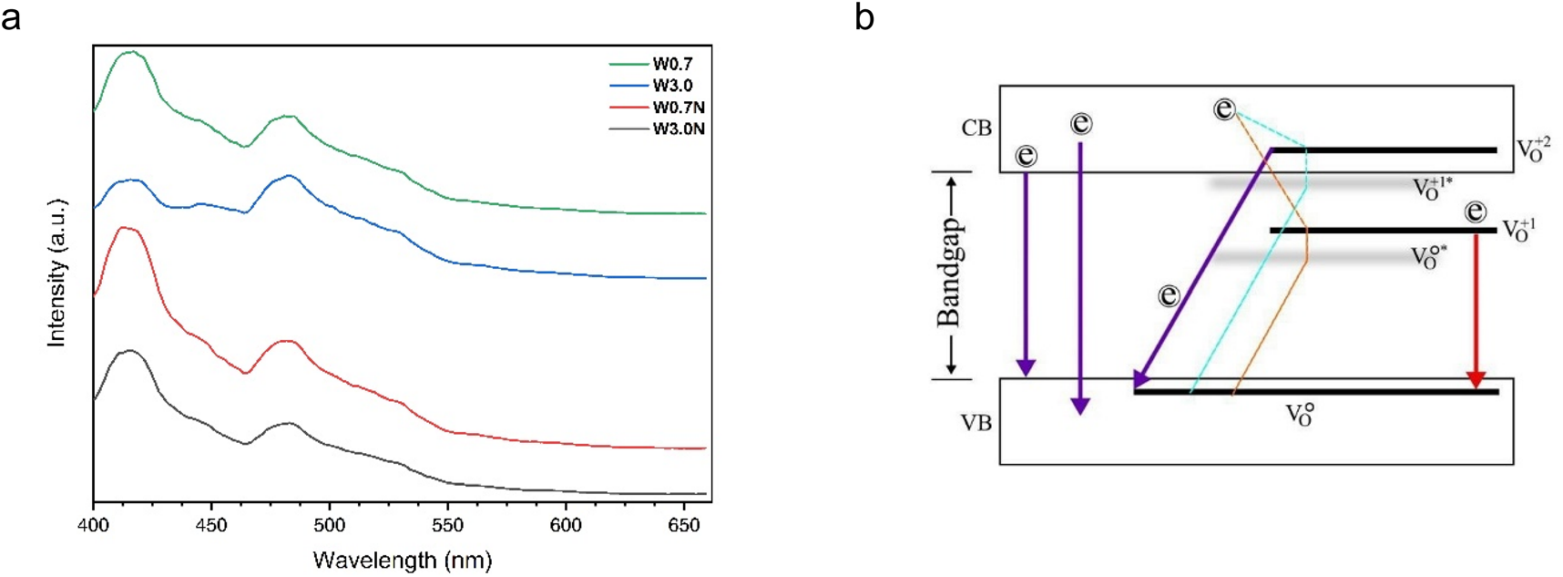
(a) PL spectra of W0.7, W3.0, W0.7N, and W3.0N, (b) energy-level diagram based on the photoluminescence spectra of WO_3_ nanostructures.

Photoluminescence spectra of the products (Fig. 3a) can be deconvoluted to five major emission peaks at 413 nm (3.00 eV), 446 nm (2.78 eV), 477 nm (2.59 eV), 512 nm (2.42 eV), and at 569 nm (2.18 eV) (as seen in Fig. S4a–d†). While the peak positions are very similar, their intensities show differences.

In tungsten oxides, the oxygen vacancies can be neutral (V^0^_o_), single charged (V^1+^_o_) or double charged (V^2+^_o_).^44^ PL emissions occur when an excited electron relaxes from the conduction band (CB) directly back to the valence band (VB), or from a charged vacancy state (V^1+^_o_ or V^2+^_o_) to the VB. Also, an electron from conduction band can relax non-radiatively into a (V^2+^_o_ and V^1+^_o_) state, forming a (V^1+^_o_ and V^0^_o_)* center.^42,45^

All four PL emission spectra have a blue emission peak at 413 nm (3.00 eV). Overall, the origin of blue emission (in the range of 413–415 nm) can be attributed to the recombination between the electron occupying the resonant defect state in the conduction band and a hole in the valence band. This electron–hole pair forms a Frenkel exciton, which is probably trapped by the resonance mode caused by the O vacancy (Table 1).^45^ The overlap of the intensity of the emission related to direct bandgap and the presence of defects significantly increases the intensity of the blue emission (in the range of 413–415 nm) for W0.7N nanorod compared to W3.0N and W0.7 (Table 1). Based on this, it appears that longer nanorods have more intense PL emission due to faster 1D crystal growth and more defects. As previously reported, Lee *et al.* showed that all three PL emission spectra have an additional blue emission peak at 437 nm (2.84 eV), and the intensity of this peak relative to the UV emission increases with the length of the nanorods.^46^ The blue emission peaks at 448 (2.77 eV) and 478 nm (2.59 eV) are associated with the decay of an electron from the unrelaxed (denoted by *) vacancy state (V^1+^_o_ and V^0^_o_)* to the VB, respectively.^42^ The energy-level diagram based on the photoluminescence spectra is shown in Fig. 3b, and PL emissions with possible transitions are summarized in Table 1.

**Table 1:** Summary of the origin of all peaks identified in the PL emission spectra of tungsten oxide nanostructures

Peak position	W3.0	W0.7	W3.0N	W0.7N
413 nm (3.00 eV)	Probably high laying resonant state arising from O vacancies	High laying resonant state arising from O vacancies	Direct band gap and high laying resonant state arising from O vacancies	Direct band gap and high laying resonant state arising from O vacancies
446 nm (2.78 eV)	Direct bandgap	Direct bandgap	Band to band (optical band gap)	Band to band (optical band gap)
477 nm (2.59 eV)	Band to band (optical band gap); and (V^0^_o_)* → V^1+^_o_ + e_VB_	Band to band (optical band gap); and (V^0^_o_)* → V^1+^_o_ + e_VB_	(V^0^_o_)* → V^1+^_o_ + e_VB_	(V^0^_o_)* → V^1+^_o_ + e_VB_
512 nm (2.42 eV)	V^0^_o_ → (V^1+^_o_)* + e_VB_	V^0^_o_ → (V^1+^_o_)* + e_VB_	V^0^_o_ → (V^1+^_o_)* + e_VB_	V^0^_o_ → (V^1+^_o_)* + e_VB_
569 nm (2.18 eV)	V^1+^_o_ → (V^2+^_o_)* + e_VB_	V^1+^_o_ → (V^2+^_o_)* + e_VB_	V_o_^1^ → (V^2+^_o_)* + e_VB_	V^1+^_o_ → (V^2+^_o_)* + e_VB_

### UV-vis diffuse reflectance spectroscopy

**Fig. 4:**
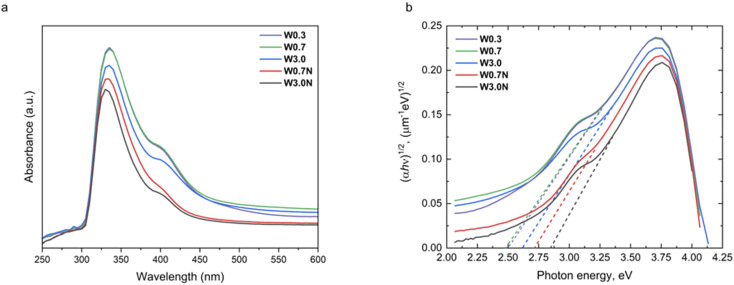
(a) The UV-Vis absorption spectra and (b) corresponding Tauc plots of W0.3, W0.7, W3.0, W0.7N, and W3.0N.

The DRS measurements of samples show different absorption edges (Fig. 4a) suggesting the band gap is indeed depending on the concentration of the precursor. To quantify the band gaps, we fit linear slopes near the absorption edge in the Tauc plots according to:^47^.(*αhν*)^*n*^ = *A*(ħ*ν* − *E*_g_)symbols *α*, *E*_g_, ħ and *ν* are the absorption coefficient, the band gap energy, Planck's constant, and the frequency of photon, respectively. The *hν* implies the incident photon energy and *A* refers to the proportional constant, while *n* is 0.5 for the indirect band gap semiconductor. From the fitting, the indirect band gap values for W0.3, W0.7, W3.0, W0.7N, and W3.0N are estimated to be 2.5, 2.5, 2.6, 2.7, and 2.9 eV, respectively (Fig. 4b). The calculated Urbach energies are 0.3 ± 0.1 eV close to those reported for WO_3_ produced by spray pyrolysis and reactive sputtering.^48,49^

### X-ray diffraction study

**Fig. 5:**
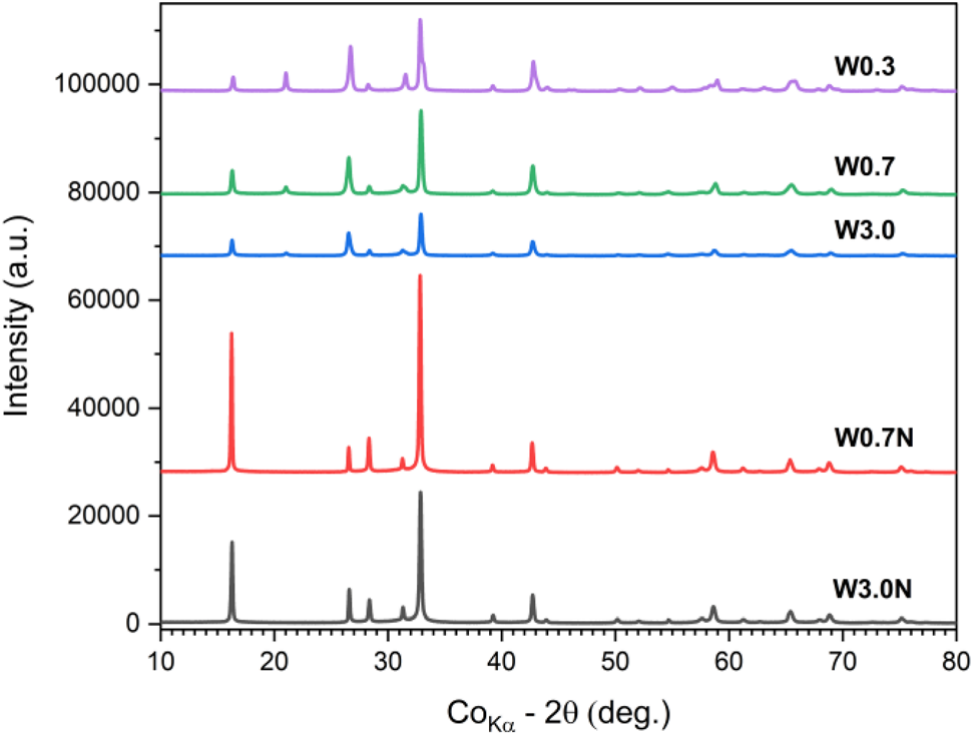
XRD patterns of W0.3, W0.7, W3.0, W0.7N, and W3.0N powder.

Since various WO_3_ phases are known to have a large variation of corresponding band gaps, it is plausible to assume that in our samples, the WO_3_ products comprise multiple phases. To confirm, we conducted XRD analyses of the powders (Fig. 5). The refined diffraction patterns show that hexagonal (ICDD no. 04-007-2322) and orthorhombic phases (ICDD no. 04-011-1708) are present in each sample, whose ratio depends on the concentration of the acid used for titrating the tungstate precursor and on the presence of sodium sulfate additive. With decreasing acid molarity from 3.0 M to 0.3 M the ∼60 wt% hexagonal phase content decreases to ∼33 wt% in the product. When sodium sulfate is added to the precursor, the hexagonal crystal content is found to be quite high (∼58 wt% for W3.0N and ∼66 wt% for W0.7N) (Table 2 and Fig. S5†).

**Table 2:** Phase composition of products

	Hexagonal (wt%)	Orthorhombic (wt%)
W3.0	59.6	40.4
W0.7	54.7	45.3
W0.3	32.7	67.3
W3.0N	58.3	41.7
W0.7N	65.5	34.5

These results well explain the trends of the measured band gaps among the different samples, since the bandgap of the hexagonal phase (2.8–3.1 eV) is considerably higher than that of the orthorhombic (2.3–2.7 eV). It is also important to note that each resolved XRD pattern (except for sample W3.0) indicates significant crystal anisotropy (Table 3) in good agreement with the morphologies of nanostructure we observed by FESEM (Fig. 1).

**Table 3:** Summary of crystal size/anisotropy of hexagonal and orthorhombic phases in the products

	Hexagonal crystal size (nm)/direction			Orthorhombic crystal size (nm)/direction		
W3.0	75/[0.513, 0.859, −0.000]	75/[−0.859, 0.513, −0.000]	53/[0.000, 0.000, 1.000]	4/[0.000,1.000,0.000]	16/[1.000,0.000,0.000]	89/[0.000,0.000,1.000]
W0.7	4/[−0.356, −0.935, 0.000]	4/[0.935, −0.356, 0.000]	130/[0.000, 0.000, 1.000]	70/[0.000,1.000,0.000]	93/[1.000,0.000,0.000]	31/[0.000,0.000,1.000]
W0.3	11/[−0.356, −0.935, 0.000]	11/[0.935, −0.356, 0.000]	1285/[0.000, 0.000, 1.000]	78/[0.000,1.000,0.000]	140/[1.000,0.000,0.000]	56/[0.000,0.000,1.000]
W3.0N	5/[−0.501, −0.865, 0.000]	5/[0.865, −0.501, 0.000]	59/[0.000,0.000,1.000]	107/[0.000,1.000,0.000]	92/[1.000,0.000,0.000]	1275/[0.000,0.000,1.000]
W0.7N	4/[−0.356, −0.935, 0.000]	4/[0.935, −0.356, 0.000]	104/[0.000, 0.000, 1.000]	139/[0.000,1.000,0.000]	87/[1.000,0.000,0.000]	280/[0.000,0.000,1.000]

### Growth mechanism

According to previous research on the growth mechanism of WO_3_, it is generally understood that in acidic environment, Na_2_WO_4_ is protonated forming H_2_WO_4_, which then participates in the formation of crystal seeds. These seeds then grow further to nanoparticles of WO_3_ by subsequent attachment of tungstate monomers on the crystal facets:^29^Na_2_WO_4_ + 2HCl + *n*H_2_O → H_2_WO_4_·*n*H_2_O + 2NaClH_2_WO_4_·*n*H_2_O → WO_3_ (crystal seed) + (*n* + 1)H_2_O → WO_3_

**Fig. 6:**
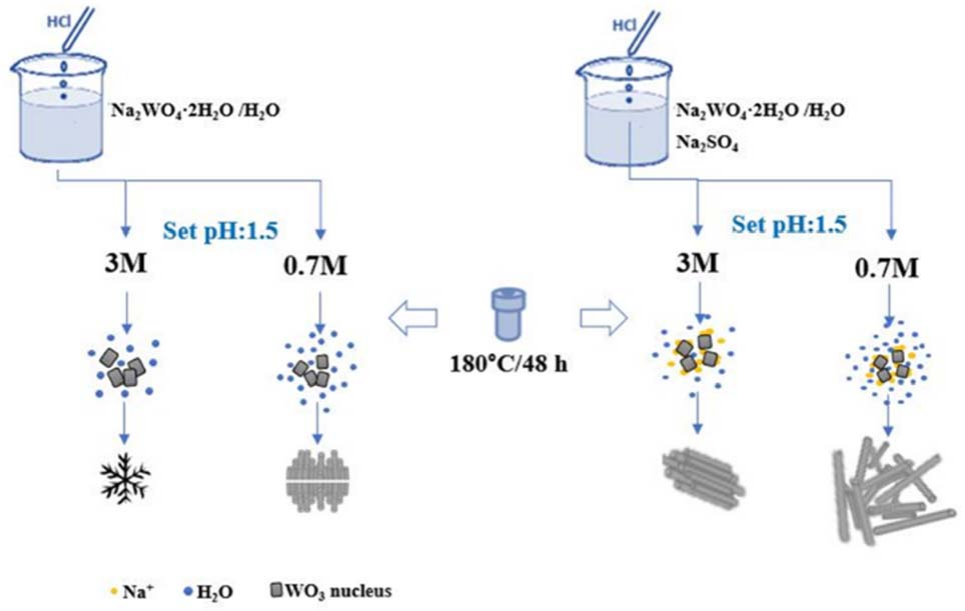
Scheme of the representation of the growth processes of WO_3_ nanostructures.

The nucleation can be heterogeneous (*e.g.* on solid surfaces^29^ or other particles) or homogeneous (as in this study). Either way, the precondition is to have a supersaturated solution from which the solute can precipitate to form the crystal seed hence lowering the Gibbs free energy. The initial shape and phase of the nucleus depends on multiple parameters (temperature, composition and concentration of the precursor) and can greatly determine the morphology of the subsequently grown nanoparticle. Without added directing agents two scenarios exist. One is the thermodynamically controlled growth, when the flux of monomers is sufficiently small so that the thermal energy of the system is not limiting the process. In this case, isotropic growth occurs. The other scenario is the kinetic growth regime, in which the supply of monomers is too rapid to enable thermodynamic equilibrium and results in the formation of anisotropic crystals.^50^ On the other hand, in the presence of capping agents, those adsorb on certain crystallographic surfaces hence inhibiting crystal growth over those thus promoting the formation of low-dimensional anisotropic structures in agreement with our study and with many other previous reports demonstrating how cations of inorganic salts and organic acids can tune the morphology of hydrothermal WO_3_ products (Fig. 6).^25,26,29,33,34,51–53^

## Conclusions

In summary, we synthesized tungsten oxide nanomaterials from precursors of different concentration but constant acidity (pH ∼ 1.5). We found that the optical and structural properties of the products were greatly influenced by concentration of sodium tungstate and sodium sulfate in the precursors. Optical band gap energy values vary between ∼2.5 eV to ∼2.9 eV for the corresponding hydrothermal growth products of mostly anisotropic crystals of mixed hexagonal and orthorhombic phases of WO_3_. The calculated Urbach energies of ∼0.3 eV and measured photoluminescence peaks between 400 and 580 nm are reasonably explained by the oxygen deficiency of crystals. Our findings indicate that the composition and structural and optical properties of formed products of hydrothermal synthesis routes may be significantly tuned even by slight changes of the precursor concentration.

## Data availability

The dataset of the article are available on Zenodo (following this DOI: https://doi.org/10.5281/zenodo.13884303).

## Author contributions

All authors contributed to the study conception and design. Material synthesis, data collection and initial analysis were done by Mohsen Zafari. The initial manuscript was completed by Mohsen Zafari and Fatemeh Shariatmadar Tehrani. The samples' structural characterization was reviewed by Krisztian Kordas and Seyed Hossein Hosseini Shokouh. Diffuse reflectance spectroscopy of the samples and Tauc plots were performed by Alexey Popov. The manuscript includes the comments of all authors, and all authors have approved the final version.

## Conflicts of interest

There are no conflicts to declare.

## Supplementary Material

RA-015-D4RA07112C-s001
