# Supplementary material for: The effect of feedstock concentration on the crystal phase, morphology, and optical properties of WO3 nanostructures
Source: RSC Adv. 2025 Jan 22;15(3):2099–105. doi: 10.1039/d4ra07112c (PMC11753408; doi:10.1039/d4ra07112c)
Supplement: RA-015-D4RA07112C-s001 [file RA-015-D4RA07112C-s001.pdf]

Supplementary Information (SI) for RSC Advances.
This journal is © The Royal Society of Chemistry 2025

Supporting Information

The effect of feedstock concentration on the crystal phase, morphology, and optical properties of WO_3 nanostructures

Mohsen Zafari[a], Fatemeh Shariatmadar Tehrani[a,], Seyed Hossein Hosseini Shokouh[b,c], Alexey A. Popov[d], and Krisztian Kordas[b]

a. Faculty of Physics, University of Semnan, P.O. Box 35195-363, Semnan, Iran
b. Microelectronics Research Unit, Faculty of Information Technology and Electrical Engineering, University of Oulu, P.O. Box 4500, FIN-90014 Oulu, Finland
c. Department of Electronics and Nanoengineering Aalto University, Tietotie 3 FI-02150, Finland
d. VTT Technical Research Centre of Finland, Kaitoväylä 1, FI-90590 Oulu, Finland.

Address correspondence to Fatemeh Shariatmadar Tehrani, f_tehrani@semnan.ac.ir; Seyed Hossein Hosseini Shokouh, seyed.hosseinishokouh@aalto.fi

A. Introduction:

Table S 1 Summary of Previous Studies on molarity and pH changes in presence or absence surfactant agents.

Creator	Used additive	Phase - Morphology	pH	Molarity
Li et al. [1]	oxalic acid ($H_2C_2O_4$)	h-WO_3 longer nanowire	0.50	12 M HCl
		h-WO_3 longer nanorods	0.80	
		h-WO_3 nanorods	1.00	
		h-WO_3 short nanorods	1.20	
Hasse Palharim et al. [2]	---	a-WO_3 phase irregularly shaped	0.50	12 M HCl
		h-WO_3 irregularly shaped	1.00	
		h-WO_3 irregularly shaped	1.50	
Shirke and Mukherjee [3]	With Na_2SO_4 / K_2SO_4	h-WO_3 nanorods	0.95	2 M HCl
		h-WO_3 nanorod bundles	1.50	
		m-$W_{18}O_{49}$ co-existed with h-WO_3 cocoon	1.76	
		m-$W_{18}O_{49}$ urchin	2.05	
		m-$W_{18}O_{49}$fishbone	2.35	
	---	o-WO_3. $(H_2O)_{0.33}$ microsphere	0.95	2 M HCl
		o-WO_3. $(H_2O)_{0.33}$ micro-disk	1.50	
		h-WO_3 nanorod bundles	1.76	
		h-WO_3 nanorod bundles	2.05	
		h-WO_3 nanorod bundles	2.35	
Nagy et al. [4]	NH_4NO_3	m-WO_3 cuboidal nanoplates	0.10	6 M HCl
		m-WO_3 + o- WO_3. $(H_2O)_{0.33}$ nanorods	0.51	
		m-WO_3 + h-WO_3 + o- WO_3. $(H_2O)_{0.33}$ nanorods + nanoneedles	1.05	
		h-WO_3 + o- WO_3. $(H_2O)_{0.33}$ nanoneedles	1.52	
		h-WO_3 nanowires	2.01	
Wang et al. [5]	Na_2SO_4 / citric acid (CA, $C_6H_8O_7$)	h-WO_3 nanoplatelets, nanoneedles, nanosheet	1.50	2–3 droplets HCl
	0.5 mol citric acid	h-WO_3 nanorods or nanoneedles diameters 5 nm to 10 nm		
	1 mol citric acid	h-WO_3 nanorods with diameter in the range of		

		50 to 60 nm and length varying from 500 nm to 1μm		
	2 mol citric acid	h-WO_3 pure nanorods (diameter of the nanorods did not further change, and length increased slightly)		
	absence of citric acid	h-WO_3 nanostructures 2-dimensional (2D) nanoplates or nanosheets 10–20 nm thick (nanoplates were up to 1 μm long and 400–500 nm wide)		
Gu et al. [6]	Rb_2SO_4 / K_2SO_4 $H_2C_2O_4$ (6.3 g)	h-WO_3 nanorod	1~1.2	3 M HCl
	0.3 g, Rb_2SO_4	h-WO_3 nanorod		
	1 g, Rb_2SO_4	h-WO_3 nanoplates packed nanowires		
	0.3 g, K_2SO_4	h-WO_3 urchinlike		
	0.5 g, K_2SO_4	h-WO_3 nanoribbons		
	1 g, K_2SO_4	h-WO_3 ribbonlike		
	1 g of Na_2SO_4	h-WO_3 cylindrical nanowires bundle		
	2 g, $(NH_4)_2SO_4$	h-WO_3 nanorods		
Peng et al. [7]	without Na_2SO_4	h-WO_3 irregular aggregation	2.00	3 M HCl
	0.125 M Na_2SO_4	h-WO_3 irregular particles, nanorods and their bundles,		
	0.25 M Na_2SO_4	h-WO_3 nanorods with diameter of 30–150 nm and length of 0.5–5 mm		
	0.50 M Na_2SO_4	h-WO_3, c- $H_2W_2O_7$ irregular lamellae and nanorods		
	1.0 M Na_2SO_4	h-WO_3, c-$H_2W_2O_7$ lamellar aggregation and few irregular particles		
	2.0 M Na_2SO_4	h-WO_3 and c $H_2W_2O_7$		

B. FE-SEM images of the unique morphology of W0.7:

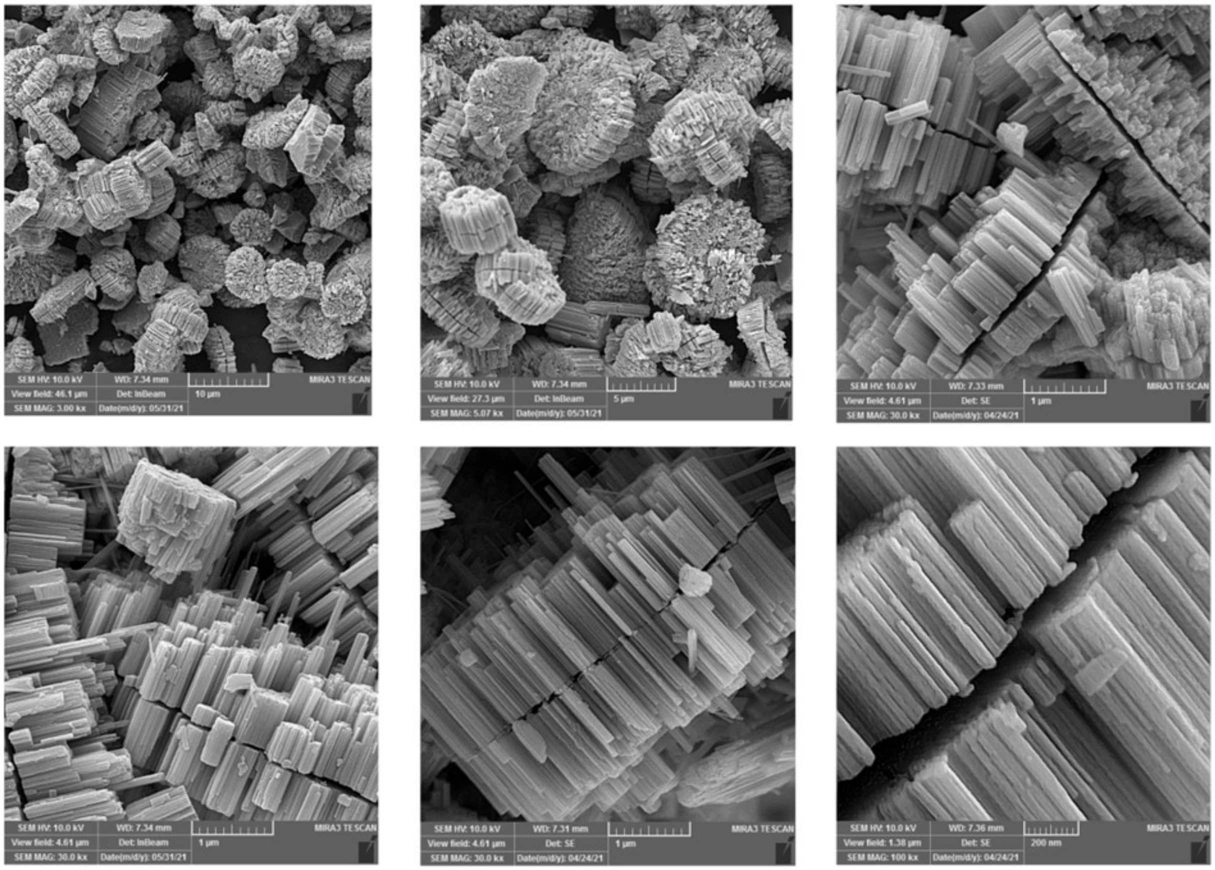

Fig. S1. FESEM image of unique morphology of sunflower-like of W0.7.

C. BET analysis:

Nitrogen adsorption-desorption isotherms of the prepared samples including W3.0, W0.3N, W0.7, and W0.7N are shown in Fig. S2. In accordance with the IUPAC classification, all of them have hysteresis loops of type H3, which is characteristic of mesoporous materials (Fig. S2) [8]. The pore size distribution and specific surface area of the products were derived using BJH and BET methods. The peaks for distribution of pore size are placed at 25.4, 6.0, 12.4, and 5.2 nm for W3.0, W0.3N, W0.7, and W0.7N, respectively. The specific surface area of samples W3.0, W0.3N, W0.7, and W0.7N is 3.9, 20.9, 8.4, and 18.6 m^2/g, respectively.

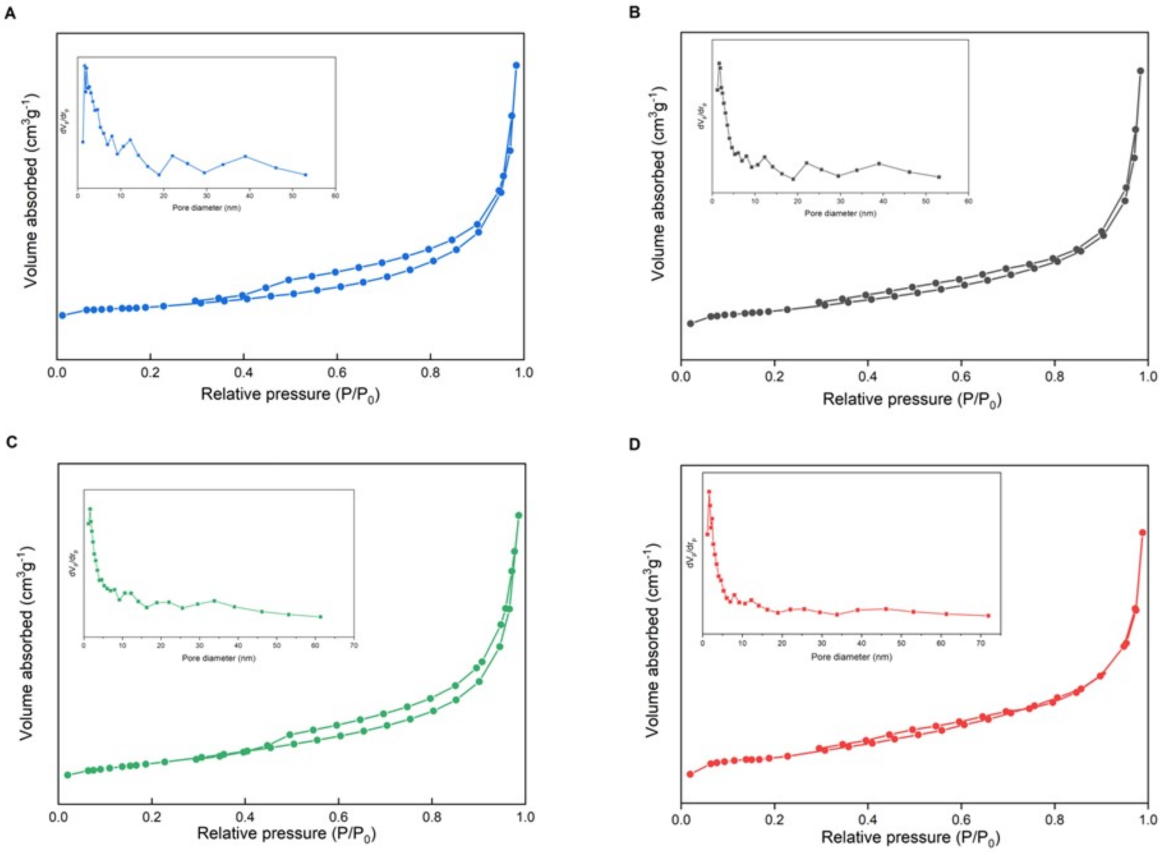

Fig. S2. N_2 adsorption-desorption isotherms and pore size distributions of (a) W3.0, (b) W3.0N, (c) W0.7, and (d) W0.7N.

D. EDX analysis:

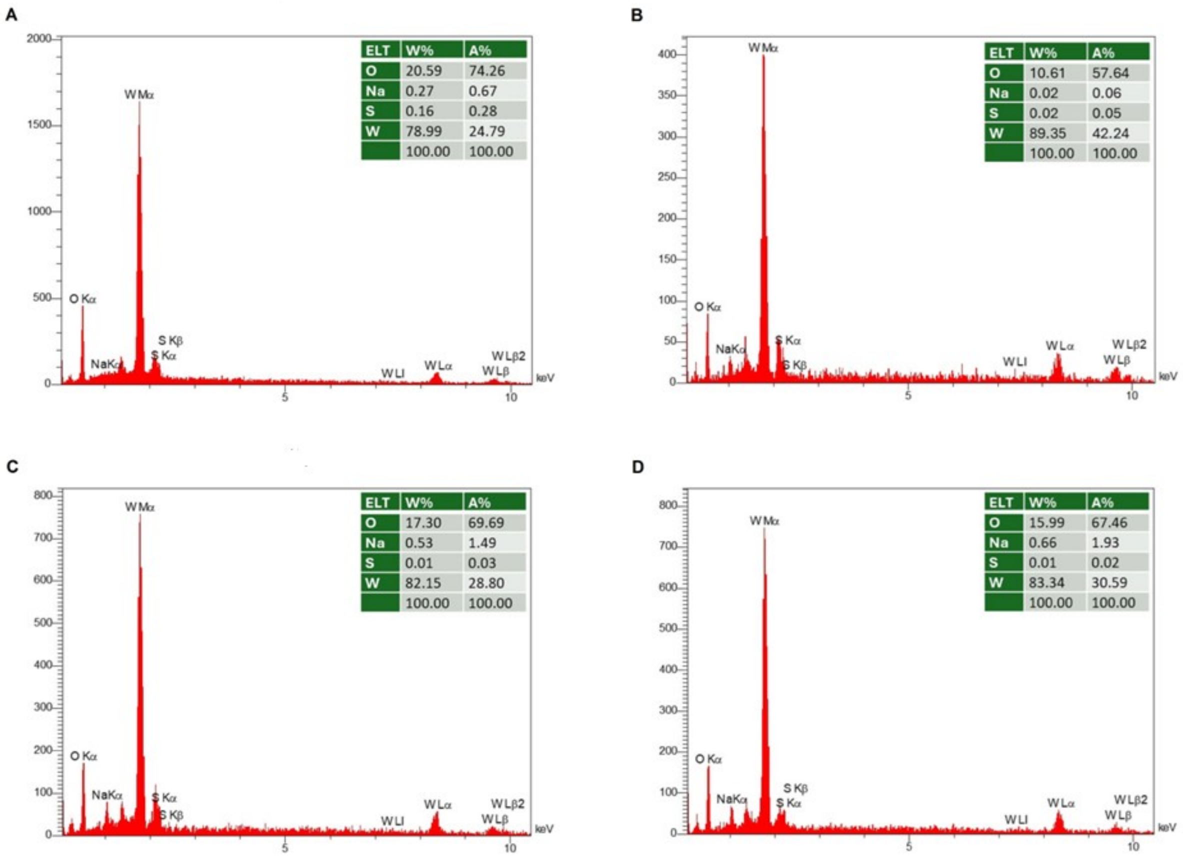

Fig. S3. EDX analysis results of (a) W3.0, (b) W3.0N, (c) W0.7, and (d) W0.7N.

.

E. FTIR and Raman analysis:

Table S 2. The list of all the relevant vibration modes observed in the FTIR spectra of nanostructures.

Wavenumber (cm^{-1})	Vibration modes	Ref.
3517.9, 3544.9, 3421.4	ν(OH)	[9,10,11]
1606.5, 1600.8	δ(OH)	[9]
1143	W-OH / δ(W-OH)	[12]
821.6, 825.4	ν(W=O)	[9]
746.4, 734.8, 711.6, 676.9, 653.8, 649.9, 644.2, 688.5, 605.6, 680.8, 690.4	ν(W=O) / ν(O-W-O)	[9,11]

Table S 3. The list of vibration modes observed in the Raman spectra.

Raman Shift (cm^{-1})	Vibration mode	Ref.
926, 932	Stretching mode W=O	[9,13]
806, 816, 817	ν(O-W-O)	[9,13]
714, 758	ν(W-O) / ν_a (antisymmetric stretch of transition metal oxide bond)	[9,13]
657, 667, 684	ν(O-W-O) / γ (O-W-O)	[9,13]
320, 324, 329	δ(O-W-O)	[13]
242, 243, 274	ν(O-W-O) / δ(O-W-O)	[13]
107, 112, 137	low-frequency phonon temperature change marker	[13]

F. Deconvoluted PL Spectra of WO_3 nanostructures:

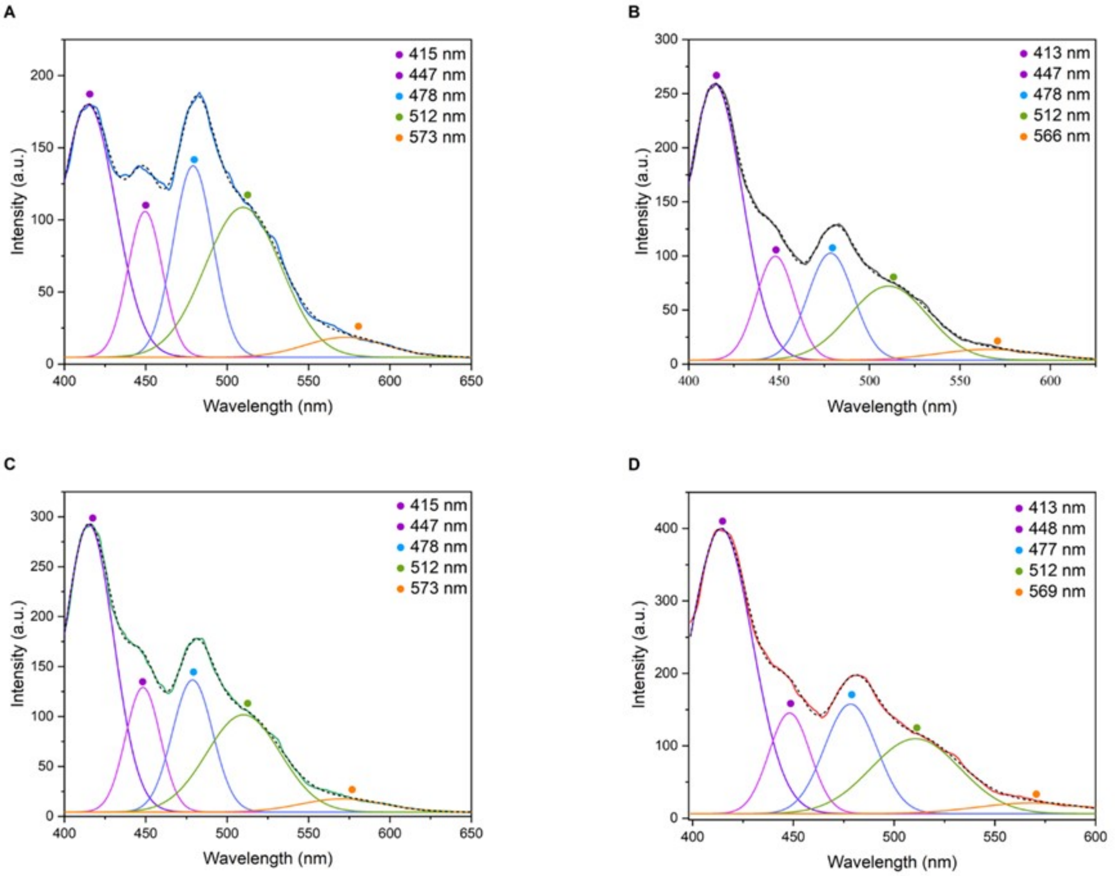

Fig. S4. Deconvoluted PL spectra of (a) W3.0, (b) W3.0N, (c) W0.7, and (d) W0.7N.

G. XRD patterns of WO_3 nanostructures:

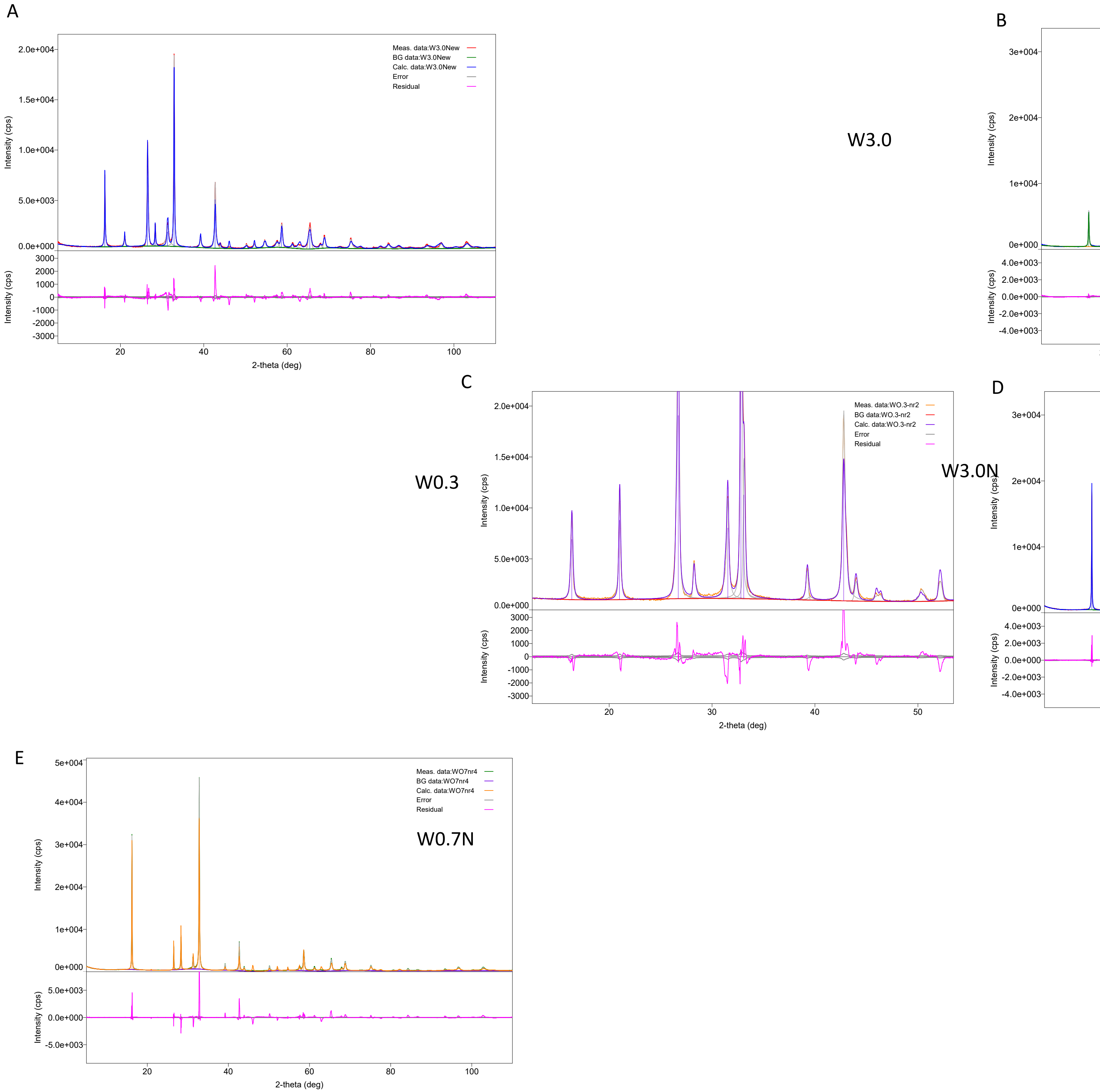

Fig. S5. XRD patterns of A) W3.0, B) W0.7, C) W0.3, D) W3.0N, and E) W0.7N along with their fitting residuals in phase analysis.

References:

[1] N. Li, T. Chang, H. Gao, X. Gao and L. Ge, *Nanotechnology*, 2019, **30**, 415601.
[2] P. Hasse Palharim, B. Lara Diego dos Reis Fusari, B. Ramos, L. Otubo and A. C. Silva Costa Teixeira, *J. Photochem. Photobiol. Chem.*, 2022, **422**, 113550.
[3] Y. M. Shirke and S. P. Mukherjee, *CrystEngComm*, 2017, **19**, 2096–2105.
[4] D. Nagy, D. Nagy, I. M. Szilágyi and X. Fan, *RSC Adv.*, 2016, **6**, 33743–33754.
[5] X. Wang, H. Zhang, L. Liu, W. Li and P. Cao, *Mater. Lett.*, 2014, **130**, 248–251.
[6] Z. Gu, T. Zhai, B. Gao, X. Sheng, Y. Wang, H. Fu, Y. Ma and J. Yao, *J. Phys. Chem. B*, 2006, **110**, 23829–23836.
[7] T. Peng, D. Ke, J. Xiao, L. Wang, J. Hu and L. Zan, *J. Solid State Chem.*, 2012, **194**, 250–256.
[8] F. J. Sotomayor, K. A. Cychosz and M. Thommes, 2018, 18.
[9] M. Gotić, M. Ivanda, S. Popović and S. Musić, *Mater. Sci. Eng. B*, 2000, **77**, 193–201.
[10] H.-F. Pang, X. Xiang, Z.-J. Li, Y.-Q. Fu and X.-T. Zu, *Phys. Status Solidi A*, 2012, **209**, 537–544.
[11] B. Gerand and M. Fjglarz, *J. Solid State Chem.*, 1987, 13.
[12] C. Hai-Ning, preparation and characterization of optical multilayered coatings for smart windows applications, University of Minho, 2005.
[13] R. F. Garcia-Sanchez, T. Ahmido, D. Casimir, S. Baliga and P. Misra, *J. Phys. Chem. A*, 2013, **117**, 13825–13831.
